# Functional team selection as a framework for local adaptation in plants and their belowground microbiomes

**DOI:** 10.1093/ismejo/wraf137

**Published:** 2025-07-02

**Authors:** Nancy Collins Johnson, César Marín

**Affiliations:** Department of Biological Sciences, Northern Arizona University, 617 S Beaver St., Flagstaff, AZ 86011, United States; School of Earth and Sustainability, Northern Arizona University, 624 S Knoles Dr., Flagstaff, AZ 86011, United States; Centro de Investigación e Innovación para el Cambio Climático (CiiCC), Universidad Santo Tomás, Av. Ramón Picarte 1130, Valdivia 5090000, Chile; Amsterdam Institute for Life and Environment, Section Ecology & Evolution, Vrije Universiteit Amsterdam, de Boelelaan 1085, Amsterdam, HV 1081, the Netherlands

**Keywords:** complex adaptive systems, cry-for-help hypothesis, host-mediated microbiome engineering, law of increasing functional information, local adaptation, mycorrhizae, niche construction theory, plant growth-promoting bacteria, plant–soil-feedback, syntrophy

## Abstract

Multicellular organisms are hosts to diverse communities of smaller organisms known as microbiomes. Plants have distinctive microbiomes that can provide important functions related to nutrition, defense, and stress tolerance. Empirical studies provide convincing evidence that in some—but not all—circumstances, belowground microbiomes help plants adapt to their local environment. The purpose of this review is to develop functional team selection (FTS) as a framework to help predict the conditions necessary for root microbiomes to generate local adaptation for their plant hosts. FTS envisions plants and their microbiomes as complex adaptive systems, and plant adaptations as emergent properties of these systems. If plants have the capacity to recognize and cultivate beneficial microbes and suppress pathogens, then it is possible for plants to evolve the capacity to gain adaptations by curating their microbiome. In resource-limited and stressful environments, the emergent functions of complex microbial systems may contribute to positive feedback linked to plant vigor, and ultimately, local adaptation. The key factors in this process are: (i) selective force, (ii) host constitution, (iii) microbial diversity, and (iv) time. There is increasing interest in harnessing beneficial microbial interactions in agriculture and many microbial growth-promoting products are commercially available, but their use is controversial because a large proportion of these products fail to consistently enhance plant growth. The FTS framework may help direct the development of durable plant-microbiome systems that enhance crop production and diminish pathogens. It may also provide valuable insights for understanding and managing other kinds of host-microbe systems.

## Introduction

The definition of an individual plant is not as simple as it seems. Innovations in high throughput molecular techniques have revealed a surprising diversity of prokaryotes, eukaryotes, and viruses inside and surrounding plant tissues [1–3]. This discovery led to the recognition of plants as holobionts composed of a host plus their microbiome, which is comprised of diverse microbial communities that can shape plant phenotypes [4, 5]. Microorganisms inhabit all plant parts, and this review will mainly focus on belowground microbiomes. Plant growth promoting bacteria and root and rhizosphere fungi (Box 1) are important components of soil-borne microbiomes that improve plant nutrition and resistance to biotic and abiotic stress [6, 7].

**Figure 1:**
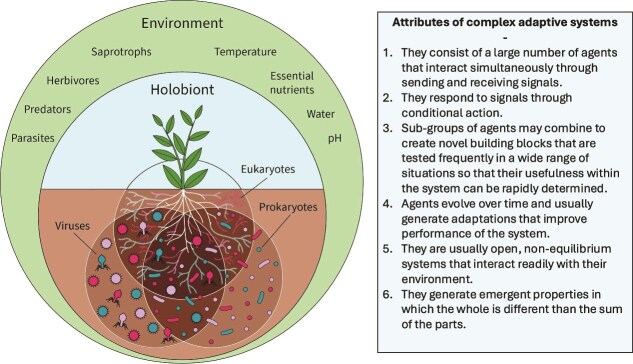
A plant holobiont is a complex system composed of a host plant plus diverse communities of interacting microorganisms both above- and belowground. The holobiont is an open system and external environmental factors (outer circle) generate selection pressures on the system. This review focuses on the root-associated microbiome comprised of interacting communities of eukaryotes (fungi, micro-invertebrates, and protists), prokaryotes (bacteria and archaea), and viruses. Functional plant-microbiome teams fit all the criteria to be considered complex adaptive systems [113, 128] composed of organisms that work together to improve plant fitness and generate adaptive traits for the host.

It is likely that vascular plants have never existed in the absence of fungal and bacterial associates [8, 9]. Fossils indicate that mycorrhizal symbioses predate the evolution of plant roots so it may be assumed that many root traits evolved to house and nurture communities of fungi and associated microorganisms [8, 9]. Arbuscular mycorrhizal (AM) symbioses occur in over 72% of all plants, including nearly all crops [10], and ectomycorrhizal (EM) symbioses are present in over 11% of plant species [10] and occur in dominant forest trees that cover an estimated 60% of the Earth’s tree stems [11]. Plants allocate 10 to 30% of their photosynthetic production to mycorrhizal fungi and an additional 5% to 21% to root exudates [12]. This tremendous investment of organic substrates belowground fosters dynamic rhizosphere communities that function like complex adaptive systems composed of host plants, associated microbes, and the surrounding environment (Fig. 1).

Box 1 Glossary of terms (underlined in the text).

- Complex adaptive systems—Dynamic systems of components that interact and adapt [113].
- Context-dependency—When the functional outcome of interactions varies with environmental conditions.
- Cry-for-help hypothesis—Predicts that stressed plants manipulate microbial communities in their rhizosphere through the release of specific compounds in root exudates that enhance populations of beneficial microbes and inhibit pathogens and herbivores [57].
- Ecological inheritance—The process by which an organism’s environment is modified by previous generations and these changes persist for future generations and impact the selection pressures they face [80].
- Ecological succession—Describes the temporal dynamics of populations and communities as well as the abiotic components of ecosystems [42, 97, 112].
- Emergent property—When individual components interact to create distinct collective properties and functions that are not manifested unless the interacting system is observed in its entirety [58].
- Functional teams—Groups of host-associated microorganisms that work together to create functions that generate adaptive traits for their host organism.
- Functional team selection (FTS) —Mechanistic framework that links evolutionary and ecological processes in space and time to account for the generation of locally adapted host-microbiome systems.
- Horizontal gene transfer—Exchange of genetic material from donor to recipient cells in organisms that are not in a parent–offspring relationship [101].
- Host-mediated microbiome engineering—Artificial selection of microbiomes through cyclical propagation of hosts and selection of the microbiome to increase or decrease certain host traits in each subsequent generation [76, 118, 121].
- Hyphosphere—Region in soil that is adjacent to and impacted by the hyphae of mycorrhizal fungi.
- Law of increasing functional information—The premise that a system will evolve if many different configurations of the system undergo selection for one or more functions [63].
- Microbe-mediated local adaptation—Enhanced host relative fitness that is partially or entirely the result of evolutionary interactions among local microorganisms [20].
- Microbe-mediated adaptive plasticity—Enhanced host relative fitness resulting from plasticity generated by interactions with local microorganisms [20].
- Multilevel selection—Occurs when natural selection operates simultaneously in at least two levels of the biological hierarchy [78].
- Niche construction theory—Explicitly recognizes that organisms modify their environment and that these legacy effects create “ecological inheritance” and contribute to evolutionary changes because, over time, they modify selection pressures on descendant organisms [79].
- Plant–soil-feedback—Interactions between plants and the soil environment that influence the growth and performance of subsequent plants. These interactions occur as plants modify the soil’s physical, chemical, and biological properties during their growth [39, 41].
- Rhizosphere—Region in the soil adjacent to and impacted by the roots of a plant.
- Rhizophagy—The process by which plants extract nutrients from microbes that live in symbiotic relationships with the plant’s roots [87].
- Syntrophy—When two (or more) species cooperatively exchange essential resources through cross-feeding [107].

Hyphae of mycorrhizal fungi extend the absorptive surface area of plant roots by several orders of magnitude [13], and associated bacteria further enhance the capability of mycorrhizal fungi to acquire resources for plant hosts. Mycorrhizal fungi do not function in isolation but actively recruit beneficial bacteria that support the symbiosis [14]. Studies have shown that hyphosphere bacteria directly contribute to the nutrition of the host plants in both AM and EM symbioses [14–16]. This hierarchically nested arrangement of symbionts within symbionts challenges traditional evolutionary paradigms that rely on a restricted definition of organismal individuality and units of selection [17]. It also challenges the traditional modeling of cooperation in the mycorrhizal symbioses as involving only the host and the fungus through direct reciprocity [18]. A better understanding of how multilevel selection generates functional plant and mycorrhizal microbiomes is needed [19]. This review explores how ecological and evolutionary processes interact across space and time to generate microbe-mediated local adaption in plants [20]. We introduce functional team selection (FTS) as a complex adaptive systems framework to test hypotheses about the mechanisms by which plants recruit and cultivate communities of rhizosphere microbes to ameliorate resource limitation and other stressors (Fig. 1).

FTS provides a spatial and temporal framework to help predict the conditions necessary to assemble microbiomes that function as collaborative teams, improving the fitness of their host plant while promoting local adaptation. The dynamic complex adaptive system perspective of FTS distinguishes it from previous models of holobiont evolution that focus on quantitative genetics of host and symbiont populations [21]. We have reviewed these previous models in Table S1 (Supplementary Information). The concept of hologenomes as hierarchically nested but not necessarily integrated host and microbiome genomes, and the view that holobiont functions are emergent properties of interactions among hosts and their microbiome were proposed over a decade ago [22, 23]. FTS builds upon these insights by hypothesizing four criteria that may integrate the forces of natural selection across levels of biological organization (aka multilevel selection), such that locally adapted holobionts are generated in constantly changing environments.

## Belowground microbiomes generate plant adaptation

There is solid evidence that microbiomes play a central role in shaping plant phenotypes and adaptive traits [24, 25]. For example, a grass capable of surviving extreme heat at Yellowstone National Park was discovered to gain thermal tolerance through its symbiotic association with an endophytic fungus, which in turn hosted a virus, and it was noted that all three players in this tripartite symbiosis were required for the grass’ survival in high-temperature geothermal soils [26]. More recently, it has been shown that plant genotype-by-microbiome interactions generate adaptive traits in upland and lowland ecotypes of a common bunchgrass [27]. Microbe-mediated adaption is a well-recognized phenomenon in plants [20]. For over four decades, research has suggested that selection pressures encountered by plants, such as drought and resource limitation, may drive the evolution of local adaptations involving AM symbioses [28, 29]. Geographic isolates of both AM and EM fungi and associated communities of microbes have been shown to improve plant fitness and vigor to a greater extent in their home environments compared to novel environments [30–33], particularly under resource limited and/or stressful conditions. Research suggests that, under some conditions and depending on the context, plants can actively select the most beneficial mycorrhizal fungi [34–37]. Furthermore, AM fungi appear to cultivate a core set of bacteria in their hyphosphere which mobilize and transfer nitrogen [38] and phosphorus [14–16] from the soil to the fungus, and ultimately, to the host plant. Adding a protist to the experimental system was shown to further increase nitrogen gained by the AM fungus [38], which illustrates how trophic diversity within microbial communities can enhance nutrient availability and, ultimately, host plant fitness.

## Plant–soil-feedback

A rich literature on plant–soil-feedback documents how plant species influence the biotic and abiotic properties of their rhizosphere soil in ways that can have positive or negative effects on the performance of subsequent plants [39, 40, 41]. Positive plant–soil-feedback occurs when rhizosphere microbes enhance the fitness of their associated plant species, which maintains the dominant plant taxa within communities [41], while negative plant–soil-feedback occurs when rhizosphere microbes inhibit the performance of their associated plant species but not other plant species in the community, which is an engine for ecological succession in natural ecosystems [39, 42] and yield decline in monoculture agroecosystems [43]. It has long been recognized that the key to harnessing soil microbiomes for more sustainable agriculture is to maximize their role in positive plant–soil-feedback and enhance microbial functions that improve plant performance and minimize functions that depress plant growth [44]. However, achieving this goal has been elusive because there is still little understanding about the mechanisms by which plants assemble and control their microbiomes to help them adapt to local environmental conditions.

## Selection in complex adaptive systems

**Figure 2:**
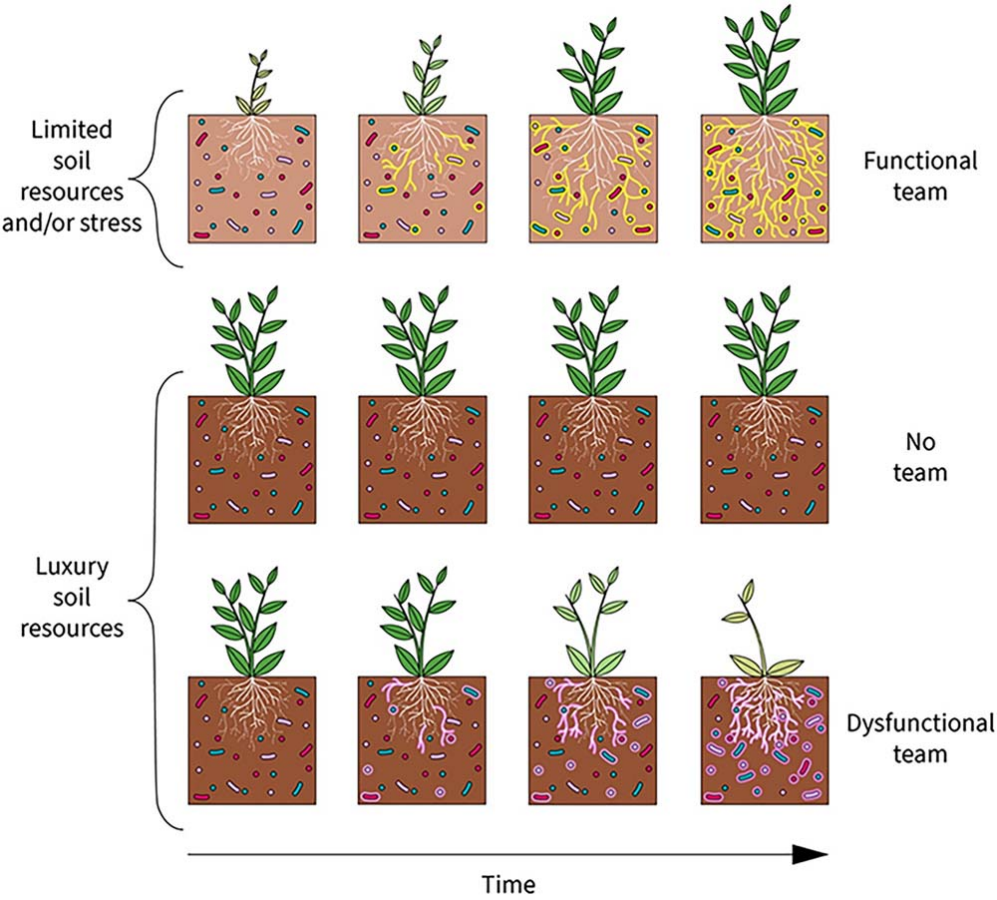
Functional team selection can drive the assembly and maintenance of communities of belowground microorganisms that improve host plant fitness when: (i) there is a selective force (e.g. limited soil resources and/or stress) that can be ameliorated by microbiome function, (ii) plant hosts can curate their microbiome (i.e. plants can selectively recruit beneficial microorganisms and deter detrimental ones), (iii) there is sufficient microbial diversity to provide adaptive functions, and (iv) there is sufficient time for a functional team to evolve (top panel). No functional team is predicted to evolve in the absence of these four requirements (middle panel). A dysfunctional team dominated by antagonistic interactions may occur when luxury supplies of essential resources and lack of environmental stress precludes selection for beneficial plant-microbe interactions. Over time, this dynamic may reduce crop yields in agricultural systems and contribute to plant succession in natural ecosystems (bottom panel).

Functional team selection provides a framework for predicting whether plant–soil-feedback will most likely be positive or negative in the *short-term* and whether microbiome-mediated local adaptation is expected to evolve in the *long-term*. It envisions a host plant and diverse communities of interacting soil-borne organisms as complex adaptive systems that are assembled and maintained through many dynamic processes in space and time (Fig. 2). Functional teams can form when a selective force such as resource limitation or stress can be ameliorated by the microbiome, the host has the capacity to selectively recruit (i.e. curate) its microbiome, and there is sufficient diversity and sufficient time for assembly and selection processes to generate a functional team of microbes. These processes can be simplified into four factors driving FTS: (i) selective force, (ii) host constitution, (iii) microbial diversity, and (iv) time (Table 1).

**Table 1:** Functional team selection is possible when a selective force can be strongly linked to traits provided by the microbiome, the host has the capacity to curate its microbiome, and there is sufficient microbial diversity, and sufficient time for community assembly and selection to generate a functional team of microbes. Examples of evidence for each of these criteria are summarized in this table.

Factors driving functional team selection	Evidence from plant holobionts
A selective force is linked to traits provided by the microbiome.	Belowground microbiomes that provide beneficial plant traits are most likely to occur in resource-limited ecosystems [46, 48, 49], stressful environments [20, 47, 115, 116, 118, 119, 126], and systems with plant pathogens [27, 59, 125].
The capacity to curate the microbiome is a heritable host trait.	Plants manipulate their rhizosphere microbiome using morphology, exudates, metabolites and microbe-microbe interactions [53, 55]. Plants integrate environmental signals with plant immunity mechanisms to fine-tune a “metabolite-based thermostat” that determines whether a plant engages with beneficial microbes or restricts pathogens [54]. Within plant species, ecotypes differ in their abilities to encourage growth of beneficial organisms and discourage the growth of antagonistic organisms in their roots and rhizosphere, including fungi [46, 52] and bacteria [50, 51]. Plant ecotypes inoculated with sympatric microbial communities often perform better than those inoculated with allopatric microbial communities [27–33, 52], indicating that over time plant ecotypes evolve the capacity to construct microbiomes with functional teams of coadapted soil organisms.
There is sufficient microbial diversity to provide beneficial functions that can be selected to improve host fitness.	Microbial diversity in soil is inversely correlated with plant disease [61]. This suggests that with sufficient microbial diversity, plants may effectively recruit and cultivate belowground microbial communities that are antagonistic to their pathogens. Diversity of soil viruses is related to plant resistance to pathogens [66–68].
There is sufficient time for the assembly and selection of functional microbiomes.	As revealed by Sewall Wright, adaptive landscapes are dynamic in space and time [111]. Ecological succession describes the temporal dynamics of the biotic and abiotic environments and negative plant–soil-feedback has been shown to be most common in early successional systems and positive plant–soil-feedback in late successional systems [42]. This finding supports the premise that sufficient time is required for the selection and accumulation of functional teams of beneficial organisms in plant rhizospheres.

Decades of empirical evidence suggest that resource-limited soil generally favors the selection and proliferation of communities of AM fungi and associated soil-borne microbes that improve plant nutrition [44–51] and drought tolerance [28, 31, 33, 41]. The vast empirical evidence of selective recruitment of beneficial microbial teams comes from ecological studies showing that plants inoculated with sympatric microbial communities often perform better than those inoculated with allopatric microbial communities [27–33, 52], particularly under stressful or resource limited conditions (Box 2). From the perspective of plant traits, this selective recruitment has been explained by changes in root morphology, metabolites, microbe-microbe interactions, and root exudates [53–55]. Future research is needed to elucidate key traits of the microeukaryotes, prokaryotes, and viruses which improve host plant performance.

Box 2 Field-based empirical evidence for local adaptation through functional team selection.

Functional team selection (FTS) articulates multilevel selection at the holobiont scale. It arose from thirty years of studies designed to understand the factors controlling mycorrhizal function in natural and agricultural systems. The critical importance of fungal-associated bacteria for mycorrhizal function has been recognized [16, 24, 129], and consequently, the “functional teams” in FTS refers to complex microbial communities composed of plant-associated fungi and all accompanying microbes (Fig. 1), including protists and other microeukaryotes [e.g. 38]. Field experiments in native grasslands show that long-term fertilization changes the species composition of arbuscular mycorrhizal (AM) fungi, and subsequent greenhouse and *in-situ* tests show that the fertilization treatments reduce the mutualistic function of AM fungi and associated microbes [48, 93]. These findings support the first premise of FTS that beneficial microbial teams arise in response to selection pressures that can be ameliorated by root-associated microbes. Experimental removal of the selection pressure (nutrient limitation) through fertilization changes the composition of the microbial community such that it is less beneficial to host plants. This discovery has sobering ramifications for efforts to harness beneficial mycorrhizal symbioses in agricultural systems because it implies that, through fertilization, farmers could inadvertently be selecting for dysfunctional microbial communities that are less beneficial or even antagonistic to crops (Fig. 2), as shown by Johnson et al. [44], Peng et al. [92], and many others (meta-analyzed in Hoeksema et al. [91]).

A reciprocal inoculation experiment comparing the functioning of mycorrhizal symbioses in genetically distinct ecotypes of the common C_4_ grass *Andropogon gerardii* from three different native prairies [46] supports the FTS assumptions that: 1) scarcity of essential nutrients can select for belowground microbiomes that enhance nutrient acquisition by plants, and 2) the fitness of both plants and AM fungi is higher in co-adapted communities of sympatric organisms compared to allopatric communities with no history of interaction. Soils at two of the experimental prairies were limited in phosphorus, and soil at the third prairie was limited in nitrogen. Formation of AM fungal hyphae in the soil, and arbuscules inside plant roots were positively correlated with P-uptake in the two P-limited sites and positively correlated with N-uptake in the N-limited site. Furthermore, the grasses grew larger and developed the most seedheads when they were grown in their home soil and inoculated with their sympatric microbiome indicating that plant fitness is higher in locally adapted combinations of plants, soil, and microbes [46].

Drought stress, like nutrient limitation, is a selection pressure that can be ameliorated by belowground microbiomes. Enhanced drought resistance has been linked to locally adapted microbiomes in ecotypes of *Bouteloua gracilis*, a common C_4_ grass in semi-arid regions [33]. A reciprocal inoculation experiment shows that sympatric microbial communities improve *B. gracilis* performance, while allopatric microbial communities depress *B. gracilis* grown under drought conditions [33]. A 3-year *in-situ* experiment that transplanted *B. gracilis* across a natural precipitation gradient further supports the FTS predictions that sympatric teams should outperform allopatric ones, and that microbial communities that function as beneficial teams are important for plant performance in stressful water limited environments but not in more benign environments [47].

Plant recruitment of beneficial microbiomes is not a universal phenomenon. In contrast to systems with resource limitations, benign environments with luxury supplies of soil resources do not favor the proliferation of beneficial communities of rhizosphere microbes, but instead, the accumulation of organisms with neutral or even antagonistic relationships with plants [24, 48, 49, 56]. These studies suggest that many plants have evolved the capacity to actively recruit beneficial teams of microbes when they experience a selection pressure that *could be ameliorated by microbial associates*, e.g. nutrient or water limitation, or pathogen pressure [50, 51, 57]. But, when there is no selection for a functional team that increases host fitness, then microbe-mediated adaptation is unlikely to evolve and instead microbiomes with commensal or even antagonistic phenotypes may be expected [49, 56] (Fig. 2). Selection pressure is not the only prerequisite for the evolution of functional teams. Host plants must have heritable traits for controlling its microbiome, there must be sufficient microbial diversity, and sufficient time for these interactions to generate functional teams that contribute to plant adaptations to local environments. This perspective accounts for the trillions of diverse rhizosphere and hyphosphere organisms that have the potential to directly and indirectly interact with each other, and with their host plant, as competitors, pathogens, commensals, and mutualists. Emergent properties of these systems of interactions [58] may either increase (positive plant–soil-feedback) or decrease (negative plant–soil-feedback) plant host performance.

Functional host-microbiome teams are analogous to sports teams composed of actively interacting players surrounded by a bench of inactive (dormant) potential players that may join or leave the team at any time. In this analogy, the purpose of the game (*aka* driver of natural selection) is to directly or indirectly support the host plant’s production of organic compounds because if the host plant does well, then populations of microorganisms associated with roots and mycorrhizal hyphae will increase, but if the host does poorly and dies, then these microbial habitats and substrates will dwindle and eventually disappear. Plant-microbiome teams compete with their neighbors for resources, are impacted by various environmental stresses, and are attacked by pathogens and herbivores. Winning teams contribute to positive plant–soil-feedback that reinforce a plant ecotype’s competitive dominance within the plant community while losing teams drive negative plant–soil-feedback and eventual replacement by different plant-microbiome teams [39, 42]. Emergent functions that are derived from the entire interaction network of plant-microbiome teams determine the success of the host plant within its local environment. As in human games, teamwork is key to winning, and teams with one or two star players can still lose the game if the whole team doesn’t work together in a coordinated fashion. The importance of a team perspective for managing plant–soil-feedbacks could explain why large-scale field inoculations that focus entirely on the introduction of a single (“star”) taxon of AM fungus fail to provide consistent benefits to crops [59, 60]. Like human teams, plant-microbiome teams may maintain taxa for different functions in the form of generalist and specialist organisms. In this regard, a high diversity of belowground communities has been observed to be positively correlated with the stability and function of plant-microbiome systems [61]. We hypothesize that this occurs because a higher diversity of microbial species increases the potential for advantageous associations in variable environments and the probability of the emergence of beneficial interactions and epistatic interactions [62] with functional significance.

## Theoretical foundation for functional team selection

Functional team selection melds evolutionary and ecological processes to encompass the totality of interactions among plants and their microbiome systems (Fig. S1, Supplementary Information). Theoretical support for FTS can be found from disparate sources. A team of planetary scientists recently posited the “law of increasing functional information” [63] to account for the universal similarities of evolving systems. It states that all evolving systems share three characteristics: (i) they form from numerous interacting components; (ii) the components can generate many different configurations; and (iii) certain configurations are preferentially selected because they display useful functions [63]. Plant-microbiome teams fulfill all three of these requirements as they are: (i) composed of a host and a network of countless interacting microbes; (ii) the microbes can interact with each other and with their host plant in a myriad of configurations; and (iii) certain configurations of plant hosts and associated microbes will function better than others in terms of increasing the productivity, survival, and reproductive success of the host plant.

The astronomical biodiversity of soil should be emphasized in the context of the first premise of the law of increasing functional information (numerous interacting components). A single gram of soil can contain more than a billion viruses [64], a billion bacteria, thousands to millions of microeukaryotes [65], and 200 meters of fungal hyphae, all belonging to several thousand species [65]. Viruses can infect all components of holobionts (plants, bacteria, and fungi) [66] and play important roles in the creation of adaptive innovations [26, 67, 68]. Furthermore, viruses function as vectors for horizontal gene transfer which allows for adaptive innovations to spread across unrelated taxa within the holobiont [69]. This tremendous abundance and diversity of organisms surrounding plant roots increases the raw material from which beneficial functions may be selected. Specific configurations of plant-microbiome systems are preferentially selected because they display useful functions, and in this case, the function is to enhance the productivity and relative fitness of the host plant.

In functional teams it is important to emphasize the importance of *functional* rather than *taxonomic* diversity. A growing literature has revealed that in many host-microbiome systems, there is surprisingly little specificity matching particular hosts with particular microbial taxa, but rather, the functions of microbial symbionts remain constant while the taxa that perform those functions vary over time and space [70–73]. This functional but not taxonomic stability of microbiomes [70] is congruent with FTS, which focuses on the functions of taxonomically plastic teams of rhizosphere organisms that directly or indirectly influence plant fitness. Lenski’s long-term evolution experiment that monitored the divergence of 12 populations of *Escherichia coli* clearly demonstrated how mutation and selection can generate diverse functional innovations from a single ancestral strain [74]. A similar genotypic and phenotypic divergence has been demonstrated from a single AM fungal spore [75]. Apply this capacity to the hyper-diverse root and hyphal associated microbes that are maintained by plant holobionts and the possibility for functional diversity is indeed expansive.

The extent to which rhizosphere microbiomes can generate myriads of functional configurations (the second premise of the law of increasing functional information) is also supported by theoretical and empirical evidence. Advances in evolutionary theory accommodate the potential for multilevel selection to generate collaborative groups of unrelated individuals [76, 77]. The disentanglement of units of selection helps elucidate evolutionary processes in plant-microbiome systems [78]. Specifically, the insight that selection, replication, and manifestation of accumulated adaptations can occur at different levels in the biological hierarchy [78], accounts for selection at the system (holobiont) level, which is intrinsic to FTS.

Another evolutionary insight called niche construction theory [79] explicitly recognizes that organisms modify their environment. These legacy effects create “ecological inheritance” [80] and contribute to evolutionary changes because, over time, they modify selection pressures on descendant organisms [79] and potentially influence the composition and function of associated microbiomes. Plant–soil-feedback is a subset of niche construction theory that focuses on plants and occurs at an ecological rather than evolutionary timeframe [81]. According to niche construction theory, plants and microbes can be considered ecosystem engineers because they influence the physicochemical properties of the soil they inhabit. As mentioned previously, plants inject enormous quantities of organic compounds belowground, which provides substrates for soil food webs and improves the water and nutrient-holding capacity of the soil [82]. Plant roots, fungal hyphae, and bacterial biofilms hold soil particles in place and generate stable aggregates that affect the amount of water and air in soils [82, 83]. Soil chemical transformations mediated by plants and microbes influence the mobility of minerals and organic compounds. This impacts soil fertility which, over time, generates abiotic feedback to the system. The classical gene-centric perspective of evolution sees the organism as a passive agent, with no influence on their environment. In contrast, niche construction and FTS assume that plants and microbes can play an active role in structuring their environment [84]. Envisioning plant holobionts as complex adaptive systems (Fig. 1), accounts for indirect effects of feedbacks with the biotic environment including the hierarchy of interactions within the holobiont as well as interactions among other local plants and animals.

In evolving systems, certain configurations are preferentially selected because they display useful functions (the third premise of the law of increasing functional information [63]). This is foundational to the FTS framework because selection by a hierarchy of organisms within the holobiont is responsible for the emergent properties of the host (*aka* holobiont phenotype). Plants and rhizosphere microbes can send signals using various chemical metabolites, which can either encourage or discourage further transactions [63, 85]. For example, studies show that plants preferentially allocate photosynthate to the most beneficial fungal symbionts [34, 35]. The level of control that plants may exert over the composition of their microbiome appears to be heritable [27, 52, 86]. The well-supported “cry-for-help” hypothesis posits that in response to stresses caused by pathogens, herbivores, pollution, drought, or other factors, plant roots release specific metabolites that ameliorate the stress through the selection or recruitment of microbes that provide beneficial functions [57]. In addition to releasing chemical signals through root exudates, plants may take an even more active role in cultivating their rhizosphere microbiome. The discovery of the rhizophagy cycle hints at the astounding level of control that plants may exert on the bacterial communities surrounding their roots [87]. The rhizophagy cycle is analogous to bacterial farming by plants in which certain bacteria enter root tips, are propagated inside root cells as wall-less protoplasts that appear to provide nutrients to the plant, and some surviving bacteria are exuded back into the soil through root hairs [87]. Through rhizophagy, plants enrich their rhizosphere with high densities of plant-selected bacterial populations ready to partner with new root growth or the roots of future offspring in the neighborhood.

Superimposed on active selection of root and rhizosphere microbes by plant hosts, is selection of hyphosphere bacteria by mycorrhizal fungi. Studies indicate that AM fungi can actively select and cultivate beneficial bacteria that facilitate nitrogen and phosphorus uptake [38, 88]. Over time, this hierarchical process of selection by symbionts within symbionts can generate teams of interdependent plant-microbiome systems that *have the potential* to function to improve host plant fitness in local environments. The key question is what factors tip the systems’ function to manifest as positive rather than negative plant–soil-feedback? Here, we introduce the context-dependency of microbiome function and stress that FTS can only select for beneficial assemblages of plant-associated microbes when cultivation of beneficial microbes is under strong selection due to resource limitation or stressful biotic and/or abiotic conditions [89–91] (Fig. 2). When predicting outcomes of FTS it is critical to remember that ***there can be no selection in the absence of a selection pressure***. For example, fertilization of formerly nutrient-limited systems removes the selection pressure for efficient uptake and conservation of nutrients and causes plants to switch from being limited by belowground resources—that can be ameliorated by a functioning team of rhizosphere microbes—to being light-limited, which cannot be ameliorated by belowground functions [48]. The phenomenon of fertilization reducing beneficial functioning of belowground microbiomes has been experimentally documented in long-term field experiments [92, 93] as well as greenhouse experiments [48, 94] (Box 2).

## Spatial legacies and ecological inheritance

Over time, perennial plant roots engineer their own physical, chemical, and biotic environment because they repeatedly explore the same spaces in the soil. Each growing season, plants inherit the biotic and abiotic legacy of soil properties from the previous season. Rhizosphere habitats are heterogenous in space and time due to seasonal variation, and different lifespans of the component organisms. Plant roots vary in the rate at which they grow and decompose, ranging from woody structural roots that may live for the entire life of a tree to fine roots that turnover within months or even weeks [95]. The spatial structure created by networks of roots and mycorrhizal fungal hyphae provides seasonally dynamic habitats for bacterial communities, which in turn, may form biofilms that offer substrates for additional microorganisms [96]. The microbial communities inhabiting different regions of the rhizosphere and hyphosphere are expected to differ considerably and provide different goods and services to host plants throughout the growing season [97]. In this regard, adaptive traits can be inherited through spatial legacies as well as genetic mechanisms [70, 80]. The FTS framework embraces these insights by recognizing the importance of ecological inheritance for *in-situ* selection of functional communities of rhizosphere microbes.

As nanoimaging technologies continue to develop, more attention should be paid to determining the detailed locations of microbes around plant roots because ecological and evolutionary processes are impacted by spatial structure [98]. Mutualistic interactions are more likely to assemble and evolve in spatially structured environments than in well-mixed environments due to the increased frequency of contact between mutually beneficial partners [99, 100]. Furthermore, spatial structure enables long-term interactions among neighboring rhizosphere organisms which may lead to the exchange of adaptive genes through horizontal gene transfer [101–103], a potentially important source of genetic diversity for evolution that should not be underestimated [104–106].

The process of syntrophy is an important driver in the assembly of plant-microbiome teams in resource-limited environments. Syntrophy, which literally means “feeding together,” occurs when microbes cooperatively exchange essential resources through cross-feeding [107]. In spatially structured environments, syntrophic partnerships may evolve over time to become obligate associations if the partner organisms become increasingly dependent on each other and lose the metabolic capabilities for independent living [108]. This phenomenon occurred in the evolution of AM fungi, which are obligate biotrophs that have lost the genes required for fatty acid and sugar biosynthesis, and consequently, they must acquire these compounds through symbiosis with a living plant host [109]. Environmental *omics* combined with confocal microscopy have revealed widespread syntrophic relationships involving diverse combinations of bacteria and archaea [107]. It is likely that functional teams are composed of many layers of syntrophic associations and that these associations may assemble and evolve *in situ* to generate communities of plants and microbes that are adapted to their local environment and to each other.

Time is a component of the FTS framework because plant holobionts are dynamic systems. Selection can occur rapidly or slowly within the rhizosphere [110], and the distinction between the ecological process of community assembly and the evolutionary process of natural selection can become fuzzy. Differences in the lifespans of plants and their microbiome organisms creates some interesting evolutionary opportunities for plants. The pace of evolutionary change is typically dictated by the generational time of organisms [111], and this could significantly limit the ability of long-lived plants such as trees to adapt to changing environments. However, if long-lived plants can gain adaptive traits from their belowground microbiome, they may respond much faster to environmental fluctuations [20, 52]. Within a single growing season, rhizosphere microbial communities can diverge substantially from adjacent soil communities [112]. Enhanced host fitness within one generation resulting from a soil-borne legacy of beneficial microorganisms has been classified as microbe-mediated adaptive plasticity to distinguish it from microbe-mediated local adaptation, which entails an evolutionary process involving enhanced host fitness that is partially or entirely the result of co-evolutionary interactions with local microorganisms [20]. Differentiating between these two phenomena may be more academic than practical, but the distinction in terms of inheritance is important because it highlights the fact that associations with local communities of microorganisms may be as beneficial to plants as genetically inherited adaptations involving host-microbiome interactions.

## Functional team selection as a hypothesis testing framework

Empirical tests of the assumptions and predictions of FTS require holistic experimental designs that impose selection pressures on the plant host and subsequent analysis of the microbiome structure (i.e. microbial community composition) and function (i.e. direct and indirect effects of the microbiome on host plant performance) in response to the selection pressure. Results from reductionist laboratory and greenhouse experiments that are missing most of the complex interactions in the system may not extrapolate well for predicting and understanding the performance of dynamic plant-microbiome systems because emergent properties can only be observed (and studied) when the whole system is investigated [58, 113]. Darwin gained insights into evolution and evidence for natural selection by studying the artificial selection of domesticated plants and animals [114]. Similarly, studies of directed evolution of microbial communities and host-mediated microbiome engineering [115, 116] can be used as experimental platforms for testing the assumptions of FTS. Specifically, studies of host-mediated microbiome adaptations can help elucidate signaling between hosts and their microbiome as well as the key forces driving the assembly and maintenance of functional teams of microbes [53]. Swenson and colleagues [76] pioneered the concept of artificial ecosystem selection as a method to select functional teams of soil microbes based on plant fitness. The key factor in these studies is that while individual plant performance is the focus of the artificial selection, the underlying goal is to cultivate collaborative microbial communities in the plant rhizosphere that enhance plant performance. This approach involves exposing multiple generations of plants to a particular selection pressure and, at the end of each growing cycle, selecting the best-performing soil-borne microbiomes to inoculate the next generation of plants [76, 115, 116]. The genetics of the host plant remains constant in these experiments so that the effects can be truly attributed to changes in the belowground microbiome. Host-mediated microbiome engineering has successfully selected belowground communities that increase leaf greenness [117], plant tolerance to drought [116, 118], and soil salinity [119], among several other traits reviewed in [115, 120, 121]. Understanding how this experimental artificial selection generates rhizosphere microbiomes that enhance plant fitness will help illuminate how multilevel selection can generate local adaptation of plant-microbiome teams in natural ecosystems.

The four drivers of FTS (selective force, host constitution, microbial diversity, and time) can help guide the design of experiments that link the community composition of microbial communities with their function. Studying the role of soil microbiomes in local adaptation across environmental gradients in natural ecosystems [46] is an excellent place to start looking for insights into the selective forces and host genetics required to create durable mutualisms among plants and their belowground microbiome. Host-mediated microbiome engineering can be implemented in both field and greenhouse settings to test hypotheses about the factors controlling microbiome assembly and maintenance of functional teams. Microbial community structure can be linked to microbiome function by coupling recent advances in metagenomics and metatranscriptomics with emerging computational techniques to reveal structural differences in mutualistic and antagonistic interaction networks [122]. Microcosm studies may be used to link the diversity of rhizosphere communities with their functions e.g. [123]. Advanced microscopy and imaging technologies can be implemented with synthetic communities [124] to study the importance of spatial and temporal structure in syntrophic interactions in the rhizosphere of plants that are experiencing resource limitation or stress. Finally, experiments that test the effects of legacies of rhizosphere microbes on host plant fitness [41] can be used to examine the pace at which functional teams can—or cannot—assemble.

## Applications for functional team selection

We argue that FTS may guide efforts of farmers and land managers to sustainably harness belowground microbiomes to reduce plant disease [50, 125], and increase plant yield, survival, and stability in the face of environmental change [126]. It will also facilitate the conservation and management of belowground microbiomes in natural ecosystems. The burgeoning plant growth promotion industry sells inoculum containing mycorrhizal fungi and plant growth promoting bacteria, but there is controversy about whether the use of these commercial products is necessary or even desirable [60]. Understanding the mechanisms by which plant microbiomes assemble, evolve, and function will inform the debate about their use in commercial inoculants in agriculture.

**Figure 3:**
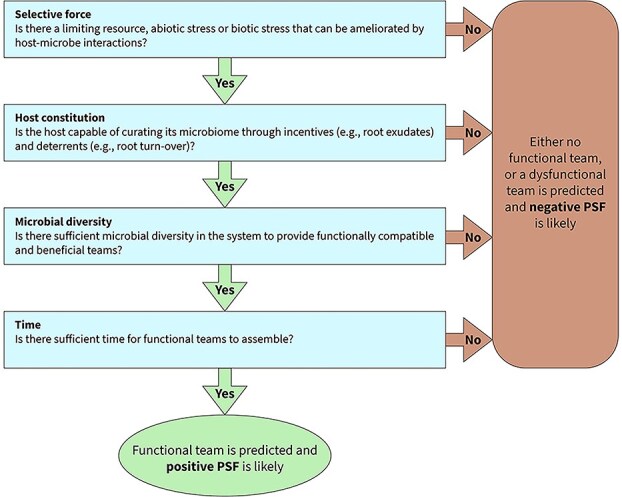
Functional team selection (FTS) helps establish the criteria required for the assembly of host-microbiome systems that improve plant performance and may generate local adaptation. The driving factors in this process are: (i) selective force, (ii) host constitution (whether the host is able to curate its microbiome), (iii) microbial diversity, and (iv) time. This decision tree illustrates how the FTS framework can be used to predict whether functional teams are expected to assemble, and in turn, whether plant–soil-feedback (PSF) will be positive and generate local adaptation, neutral with no functional team, or negative with a dysfunctional team that may contribute to plant succession or yield decline in crops.

A critical consideration in the commercial applications of plant-growth promotion products is the fact that the influence of mycorrhizae and associated bacteria on plant fitness is usually context-dependent, meaning the same communities of microorganisms can have beneficial, neutral, or even detrimental effects on host plant performance [94]. To harness belowground microbiomes for their beneficial effects may require careful consideration of the on-going selection pressures that are experienced by the rhizosphere community. Before investing in expensive commercial products to enhance crop production, farmers should answer the question—***is it even possible to expect positive plant–soil-feedback*** (Fig. 3)? Conventional agricultural practices involving fertilizers and tillage appear to inadvertently select for less mutualistic or even parasitic AM fungi and associated microbes [44, 92]. Effective management of belowground microbiomes should begin with an assessment of the biotic and abiotic stresses that can be ameliorated by a functional team of microorganisms. If there is no resource limitation or pathogen pressure, then the likelihood of observing a beneficial outcome of inoculation is slim [50].

## Next steps

Historically, the focus of microbiology was on individual, culturable organisms, and the focus of evolutionary ecology was on genetically heritable traits within populations, but now we have the technical and computational ability to investigate the evolution of complex adaptive systems [58]. High throughput technologies have revealed an unexpected universe of microbial diversity, and the extended evolutionary synthesis supports a multilevel view of natural selection to include collaborative teams of unrelated organisms [78] and feedback with the environment [40, 79]. Host-mediated microbiome engineering experiments support the view that teams of beneficial soil-borne microbes can be selected based on their ability to improve plant performance in stressful and resource-limited environments [115, 116, 118]. FTS builds upon these insights and offers a holistic framework for studying the functioning of plant microbiomes. The sports analogy helps to illustrate how teams of unrelated organisms can be selected based on their ability to work together to improve the fitness of a host plant. The next step for harnessing the benefits of belowground microbiome teams in human-managed systems is to elucidate the mechanisms that generate durable positive plant–soil-feedback.

“Consilience” describes the melding of multiple perspectives through the lenses of disparate disciplines [127]. To effectively select and engineer beneficial plant-microbiome teams that are durable in a constantly changing environment will require transdisciplinary teams of researchers in natural and computational sciences that can span the vast spatial and temporal scales experienced by plant-microbiome systems. Quantum mechanics coupled with environmental omics may help detect and understand molecular signals transmitted and received at atomic and sub-atomic scales. Plant pathology and soil science can be coupled to better understand the mechanisms driving plant–soil-feedback at organismal and community scales. Blockchain data bases coupled with field studies of microbial dynamics in ecological succession may help track legacies of niche construction over years to decades. Although the FTS framework was developed to understand the evolution and functioning of plants and their belowground microbiome, it is highly possible that this framework may also be applicable to other types of holobionts. Scientific consilience can help us better understand and manage all kinds of microbiome teams that provide essential functions for humans.

## Supplementary Material

SI_Figure_S1_wraf137

SI_Table_S1_wraf137

## Data Availability

Data sharing is not applicable to this article because no data sets were generated or analyzed.
