# Supplementary material for: Functional team selection as a framework for local adaptation in plants and their belowground microbiomes
Source: ISME J. 2025 Jul 2;19(1):wraf137. doi: 10.1093/ismejo/wraf137 (PMC12406698; doi:10.1093/ismejo/wraf137)
Supplement: SI_Figure_S1_wraf137 [file si_figure_s1_wraf137.pdf]

Supplementary Figure S1

Nancy Collins Johnson and César Marín

Functional team selection is built from many well established ecological and evolutionary processes across a hierarchy of spatial scales, from cells to communities (**Figure S1**). At the cellular and rhizosphere scale, horizontal gene transfer (Soucy et al., 2015) and syntrophy (D'Souza et al., 2018) generate subgroups of interacting microbes with potentially useful functions. 'Cry-for-help' (Rolfe et al., 2019) and rhizophagy (White et al., 2018) processes establish the capability of individual plants to select and cultivate particular groups of microorganisms that display beneficial functions. Microbe-mediated local plasticity and adaptation provide evidence linking microbiome function to plant acclimation and adaptation (Petipas et al., 2021). Niche construction (Odling-Smee 2024) and plant-soil-feedback (Bever et al., 1997; De Vries et al. 2023) connect plant holobionts with biotic and abiotic selection pressures in their community. Multilevel selection (Suárez and Lloyd 2023) explains how natural selection occurs simultaneously across all the interacting components of plant holobionts to generate functional teams that may compete with other functional teams that coexist in the same location. Adding a temporal dimension to the community scale, one may observe that functional teams may stabilize the composition of climax communities while dysfunctional teams may be important drivers of successional change (Bever et al., 1997; Kardol et al. 2006).

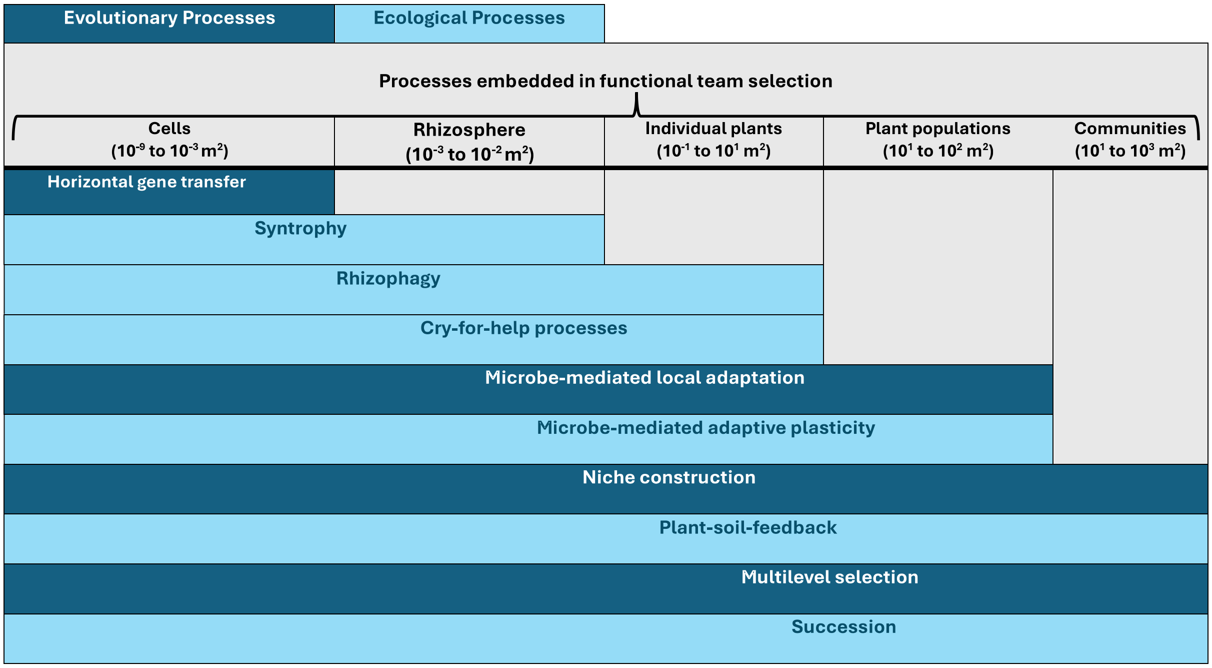

References

Bever JD, Westover KM, Antonovics J. Incorporating the soil community into plant population dynamics: The utility of the feedback approach. *J Ecol* 1997;**85**:561. doi:10.2307/2960528

De Vries F, Lau J, Hawkes C, Semchenko M. Plant–soil feedback under drought: does history shape the future? *Trends Ecology Evol* 2023;**38**:708-718. doi:10.1016/j.tree.2023.03.001

D'Souza G, Shitut S, Preussger D, Yousif G, Waschina S, Kost C. Ecology and evolution of metabolic cross-feeding interactions in bacteria. *Nat Prod Rep* 2018;**35**:455-488. doi:10.1039/c8np00009c

Kardol P, Martijn Bezemer T, Van Der Putten WH. Temporal variation in plant–soil feedback controls succession. *Ecol Lett* 2006;**9**:1080-1088. doi:10.1111/j.1461-0248.2006.00953.x

Odling-Smee J. *Niche construction how life contributes to its own evolution*. Massachusetts: MIT Press Cambridge, 2024.

Petipas RH, Geber MA, Lau JA. Microbe-mediated adaptation in plants. *Ecol Lett* 2021;**24**:1302-1317. doi:10.1111/ele.13755

Rolfe SA, Griffiths J, Ton J. Crying out for help with root exudates: adaptive mechanisms by which stressed plants assemble health-promoting soil microbiomes. *Curr Opin Microbiol* 2019;**49**:73-82. doi:10.1016/j.mib.2019.10.003

Soucy SM, Huang J, Gogarten JP. Horizontal gene transfer: building the web of life. *Nat Rev Genet* 2015;**16**:472-482. doi:10.1038/nrg3962

Suárez J, Lloyd EA. *Units of selection. Elements in the Philosophy of Biology*. Cambridge: Cambridge University Press, 2023.

White JF, Kingsley KL, Verma SK, Kowalski KP. Rhizophagy cycle: An oxidative process in plants for nutrient extraction from symbiotic microbes. *Microorganisms* 2018;**6**:95. doi:10.3390/microorganisms6030095
