# Supplementary material for: Functional team selection as a framework for local adaptation in plants and their belowground microbiomes
Source: ISME J. 2025 Jul 2;19(1):wraf137. doi: 10.1093/ismejo/wraf137 (PMC12406698; doi:10.1093/ismejo/wraf137)
Supplement: SI_Table_S1_wraf137 [file si_table_s1_wraf137.pdf]

Supplementary Table S1
Comparison of Functional Team Selection with holobiont and hologenome models

Nancy Collins Johnson and César Marín

Functional Team Selection (FTS) is a conceptual model for understanding and studying how in some circumstances, hosts can assemble and curate their microbiomes to create adaptive phenotypes. Other models have been proposed to understand various aspects of holobionts, and it is useful to compare FTS with these models and highlight their agreements and differences. **Table S1** compares FTS with the classical holobiont model (Rosenberg et al. 2007), the hologenome concept (Bordenstein and Theis 2015; Theis et al. 2016), the community genetics holobiont model (Lloyd and Wade 2019), the hologenome stability of traits/reconstitutor model (Suárez 2020; Veigl et al. 2022), and the host-orchestrated species sorting model (Roughgarden 2023). Some components of FTS overlap with these models, but most components of FTS are not considered in the other models. In this regard, FTS provides important new insights in understanding the factors that control whether microbiomes benefit or harm their hosts. Envisioning plant holobionts as dynamic complex systems that provide adaptive functions for the host is a key difference between FTS and earlier holobiont and hologenome models that are primarily based on quantitative genetics.

References

Bordenstein, S.R., Theis, K.R. (2015) Host biology in light of the microbiome: Ten principles of holobionts and hologenomes. *PLoS* 13(8):e1002226. Doi:10.137/journal.pbio.1002226

Lloyd, E., A., Wade, M., J., (2019) Criteria for holobionts from community genetics. *Biological Theory* 14:151-170.

Rosenberg, E., Kellogg, C. A., & Rohwer, F. (2007). Coral microbiology. *Oceanography*, 20(2), 146-154.

Roughgarden, J. (2023). Holobiont evolution: Population theory for the hologenome. *The American Naturalist,* 201(6), 763-778.

Suárez, J. (2020). The stability of traits conception of the hologenome: An evolutionary account of holobiont individuality. *History and Philosophy of the Life Sciences* 42 (1):1-27.

Theis, K. R., Dheilly, N. M., Klassen, J. L., Brucker, R. M., Baines, J. F., Bosch, T. C., ... & Bordenstein, S. R. (2016). Getting the hologenome concept right: an eco-evolutionary framework for hosts and their microbiomes. Msystems, 1(2), e00028-16.

Veigl, S. J., Suárez, J., & Stencel, A. (2022). Rethinking hereditary relations: the reconstitutor as the evolutionary unit of heredity. *Synthese*, 200(5), 367.

Table S1. Similarities and differences between Functional Team Selection (FTS) and other holobiont models.

Model	FTS	Classical holobiont model	Hologenome concept	Community genetics holobiont model	Stability of traits/ Reconstitutor	Host-Orchestrated Species Sorting
**Reference(s)**	This article	(Rosenberg et al. 2007)	(Theis et al. 2016; Bordenstein and Theis 2015)	(Lloyd and Wade 2019)	(Suárez 2020; Veigl et al. 2022)	(Roughgarden 2023)
**Accounts for ecological inheritance from spatial legacies.**	Yes	No	Yes (environmental filtering)	No	?	No
**Assumes context dependency and accounts for spatial and temporal dynamics in microbiome functioning.**	Yes	No	Yes	No	No	No
**Considers holobiont traits as an emergent property of the host-microbiome system.**	Yes	No	Yes	?	Yes	Yes
**Accounts for hierarchically nested symbionts within symbionts (hosts within hosts).**	Yes	No	No	No	No	No
**Provides a framework to predict when microbiomes will be beneficial or detrimental to their host.**	Yes	?	?	?	?	Yes
